# Evaluation of strategies for increasing response rates to postal questionnaires in quality control of nasal septal surgery

**DOI:** 10.1186/s13104-017-2516-x

**Published:** 2017-06-02

**Authors:** Merete T. Egeland, Magnus Tarangen, Olga Shiryaeva, Caryl Gay, Liv K. Døsen, Rolf Haye

**Affiliations:** 1 Department of Quality, Lovisenberg Diakonale Hospital, Postboks 4970, Nydalen, 0440 Oslo, Norway; 2 Department of Oto-Rhino-Laryngology, Lovisenberg Diakonale Hospital, Postboks 4970, Nydalen, 0440 Oslo, Norway; 3 Faculty of Medicine, University of Oslo, 0372 Oslo, Norway

**Keywords:** Nasal surgical procedures, Outcome assessment, Visual analogue scale, Surveys and questionnaires

## Abstract

**Background:**

Postal questionnaires are often used to assess the results of nasal septoplasty, but response rates vary widely. This study assesses strategies designed to increase the response rate.

**Methods:**

Postoperative questionnaires using visual analogue scales (VAS) for nasal obstruction were mailed to 160 consecutive patients alternately allocated to one of two groups. Group A received the questionnaire in the usual manner and group B received a modified cover letter with hand-written name and signature and a hand-stamped return envelope.

**Results:**

Of the 80 patients in each group, 47 (58.8%) in group A and 54 (67.5%) in group B returned the questionnaire (p = 0.25). There were no age or gender differences between the groups, nor did the pre- and postoperative VAS scores differ between the groups.

**Conclusion:**

The strategies used in this study increased the response rate to postal questionnaires by 8.7% points, but this was not a statistically significant or clinically meaningful improvement.

## Background

Questionnaires are commonly used in quality control of nasal surgery [1]. Response rates to postal questionnaires vary widely from 47 to 98% [2–5]. We use the Nasal Surgical Questionnaire (NSQ) [6], which has both a preoperative and a postoperative version, for ongoing evaluation of the nasal septoplasty performed in our hospital. The monthly response rate to the postal questionnaires usually varies between 64 and 67%, but in some months, it has been as low as 58%. A Cochrane review [7] evaluated a variety of methods to increase return rates of surveys and postoperative inquiries, such as the use of hand-written patient names, physician signatures, and address on envelopes; statements in the cover letter about the confidentiality of the patient’s responses and an obligation to respond; relevance, sensitivity and order of the questions; size and lay-out of the questionnaire; use of regular or first class stamps; university sponsorship; offering pecuniary and other incentives; and employing pre-notification and reminders. Although there is no consensus as to the minimum response rate required to yield accurate results, it is desirable to achieve the highest response rate possible while minimizing costs. Thus, this study was initiated to determine whether implementing five of these strategies in our quality control of nasal septal surgery could increase our response rate from the usual 65–85%, without an undue increase in expenses.

## Methods

The study was approved by the Ethics committee of Lovisenberg Diakonale Hospital. The study sample consisted of patients operated with septoplasty with or without surgery to the inferior turbinate between March and September 2015. Patients completed the preoperative version of the NSQ in the morning on the day of surgery. The postoperative version of the NSQ was mailed to each patient 5.5 months post-surgery. The one-page questionnaire contains 16 horizontally arranged questions, with the most relevant to the patient placed at the top. Sensitive and open-ended questions are avoided. The NSQ contains separate visual analogue scales (VAS) assessing nasal obstruction during the day, at night and during exercise. Each VAS has a 10 cm line, with the left end of the line representing no obstruction (numbered 0) and the right end of the line representing complete obstruction (numbered 10). The patients are asked to rate their nasal obstruction on each of the three scales with a vertical line. The VAS score represents the distance in mm between their vertical line and the left end of the scale. They are also asked retrospectively to rate their perception of improvement in obstruction following surgery as: completely, substantially, or somewhat improved, unchanged or worse. For other nasal symptoms and therapies, 4-point Likert scales are used with the following response categories: none, mild, moderate, and severe/daily symptoms/daily use. In addition, questions about smoking habits and allergies are included. In this study, all patients received the same questionnaire. We allocated every second patient to group A (standard routine), which received the usual cover letter with the name of the patient printed at the top and the signature of the surgeon copied onto the letter, the patient’s name and address visible in the envelope window and a printed stamp on the return envelope. The other patients were allocated to group B (modified routine), which received a modified cover letter with the name of the patient hand-written at the top and with the hand-written signature of the surgeon. We also added an assurance that the medical information obtained would be handled confidentially. The patient’s name and address were hand-written on a standard envelope (without a window) and the return envelope was stamped with a regular postage stamp. 3 weeks later a reminder was mailed to those who had not yet returned their questionnaire. The reminder contained the same cover letter and the same questionnaire as the patient received initially. A sample calculation indicated that at least 77 patients per group would be needed to detect an increase in the response rate from 65 to 85%, assuming power of 80% and an alpha level of 0.05. A larger sample would yield statistical significance for a smaller increase in response rate, but a 20% point increase was considered the minimum needed to justify the additional resources required to implement the modified routine.

### Statistical analysis

All analyses were conducted using SPSS version 22 (IBM Corp, Armonk, NY). Descriptive statistics (means and frequencies) were used to summarize sample characteristics and questionnaire responses. Independent sample *t* tests were used to compare responders on continuous variables. Mann–Whitney U test was used to test for group differences on ordinal variables. Chi square tests were used for group comparisons on categorical variables. A significance level of p < 0.05 was used for all analyses.

## Results

From October 2015 to April 2016 the postoperative version of the Nasal Surgical Questionnaire (NSQ) was mailed to the 160 patients (80 assigned to each group) who had been operated with nasal septoplasty with or without turbinectomy. Forty-seven (58.8%) of the 80 patients in group A (standard routine) and 54 (67.5%) of the 80 patients in group B (modified routine) returned the NSQ (p = 0.25). This increase in response rate of 8.7% points did not reach the 20% point threshold required for statistical significance in a sample of this size.

There were no significant age or gender differences between the groups (Table 1). There were also no group differences in self-reported allergy or smoking habits. Comparing the groups, no differences were found in the ratings of obstruction during the day, at night or during exercise for preoperative, postoperative and change in VAS scores (Table 2). The postoperative retrospective rating of improvement in nasal obstruction showed no statistically significant differences between the groups (Table 3).

**Table 1:** Demographic comparisons by group

Demographic characteristic	Total N = 160	Group A (standard) N = 80	Group B (modified) N = 80	p value
Age, mean ± SD	35.3 ± 12.0	34.1 ± 11.3	36.5 ± 12.7	0.21*
Gender, n (%)				0.18**
Females	53 (33.1)	31 (38.8)	22 (27.5)	
Males	107 (66.9)	49 (61.2)	58 (72.5)	

* Independent sample *t* test

** Chi square test

**Table 2:** Group comparisons of preoperative, postoperative and change in VAS obstruction scores among patients who completed both the preoperative and the postoperative questionnaires

VAS for nasal obstruction	Total N = 99 Mean ± SD	Group A, N = 47 Mean ± SD	Group B, N = 52 Mean ± SD	p value*
Day				
Preop	60.0 ± 21.5	60.4 ± 21.1	59.7 ± 22.2	0.85
Postop	23.9 ± 20.6	26.7 ± 20.5	22.9 ± 21.0	0.67
Change	35.4 ± 26.5	31.1 ± 23.9	38.2 ± 27.9	0.31
Night				
Preop	73.0 ± 19.7	71.9 ± 21.2	74.8 ± 19.1	0.66
Postop	29.5 ± 24.9	34.0 ± 24.9	29.0 ± 24.4	0.59
Change	43.8 ± 26.8	38.0 ± 27.7	45.7 ± 26.2	0.51
Activity				
Preop	64.9 ± 25.5	68.1 ± 23.8	62.2 ± 27.3	0.27
Postop	26.2 ± 22.9	32.9 ± 24.4	23.1 ± 21.1	*0.03*
Change	39.8 ± 27.4	35.3 ± 29.6	41.4 ± 24.4	0.42

*Group A* standard routine, *Group B* modified routine

Italic p value is statistically significant (p < 0.05)

**Table 3:** Group comparison of retrospective rating of postoperative improvement in nasal obstruction

Retrospective rating of postoperative improvement	Total N = 99 (%)	Group A, N = 47 (%)	Group B, N = 52 (%)	p value*
Completely improved	13 (13.1)	6 (12.8)	7 (13.5)	p = 0.45
Substantially improved	51 (51.5)	22 (46.8)	29 (55.8)	
Somewhat improved	32 (32.3)	18 (38.3)	14 (26.9)	
Unchanged	3 (3.0)	1 (2.1)	2 (3.8)	
Worse	0	0	0	

*Group A* standard routine, *Group B* modified routine

* Mann–Whitney U

## Discussion

This study evaluated the implementation of five strategies for increasing response rates to our postal questionnaire for quality control of nasal surgery. The results indicated a modest increase of 8.7% points. However, because this study was only designed to detect a larger difference of 20% points, this modest improvement was not statistically significant. This modest increase would likely have been statistically significant in a larger sample, but even with this statistical significance, it would have to be considered whether the additional resources required for the modified routine are justified to obtain such a small increase in response rate.

The patients were allocated to either the standard routine or a modified routine by alternating the group assignment for each consecutive patient. There were no group differences in age, gender or rating of nasal obstruction, suggesting that the randomisation was adequate.

There are several articles reviewing methods to increase response rates to health related questionnaires [7–12], such as use of incentives, pre-notification, reminders, importance and sensitivity of questions, size and lay-out of questionnaires and cover letters, mention of obligation to respond, university sponsorship and different mailing procedures.

Pecuniary incentives, whereby every respondent receives a monetary benefit, have been shown to be very effective in increasing response rates [7–10]. However, our hospital would not consider such an option as we perform quality control in many fields of surgery on an on-going basis whereby, pecuniary incentives would be cost prohibitive as a standard routine.

Changes to the questionnaire itself have also been found to be effective for improving response rates [7]. Shorter compared to longer questionnaires increased the response rate by about 50% and placing the most relevant questions first by about 25%. Using factual questions improved response by about 25% and interesting questions doubled the response, whereas open-ended questions reduced the response rate by 50%. Horizontal organisation of response options was better than vertical organisation. One review [8] found shorter questionnaires to increase response by 9% while another [9] found them to be only marginally better than longer questionnaire. We have avoided open ended questions which reduce response rates. Given that these strategies had already been implemented in our NSQ, their effectiveness could not be evaluated in the present study.

Modifications to the cover letter have also been shown to increase response rates [7]. An assurance of confidentiality, a hand-written instead of a printed signature by the investigator, and a hand-written instead of a printed address label were each found to increase response rates by about 25%. These measures were not considered in the other reviews. These measures were implemented in the cover letter sent to group B (modified routine). Mentioning an obligation to respond to the hospital’s questionnaire was found to increase response in prior studies. However, we believed that our patients would not respond positively to this strategy, and therefore did not implement it in the present study.

Use of regular stamps instead of printed stamps on outgoing mail has not been shown to influence response rate. However, regular instead of printed stamps on the return envelopes have been shown to increase response rates by about 25% [7]. Therefore, in our modified routine we used ordinary stamps only on the return envelopes.

The response rate to the NSQ using the standard routine was lower in this study than we usually obtain. Mailing of questionnaires is suspended during vacations and holidays. During the study period, mailing was suspended from December 15 to January 2 and accumulated questionnaires were posted after this period. However, it is possible that pre-holiday activity even before December 15 may have reduced attention to the NSQ. However, this would have influenced both groups equally as the mailings were done in parallel, and thus, does not explain the small difference in response rates between the groups.

In this study, we mailed the NSQ to 80 patients according to our standard routine (group A) and to another 80 patients using the modified routine (group B). There was a modest increase, albeit not statistically significant, in the response rate to the postal questionnaires of 14.9 or 8.7% points (from 47 patients in group A to 54 in group B). As we had combined several strategies that were individually known to be effective in increasing response rates, we had expected a larger increase in the response. Furthermore, there was an increase of 10% in the time required to prepare the mailings using the modified compared to the standard routine. The mailing cost was increased by 50%. This increase in cost might be acceptable if the return rate had been higher.

Several additional strategies might be considered to further increase response rates. Although our patients are advised that they will receive a questionnaire 6 months after the operation, we should consider sending an SMS pre-notification either for the primary questionnaire or for the reminder. This would, however, add to the cost. One might also consider applying for university approval/sponsorship.

Studies about the effectiveness of reminders in increasing response rates have yielded varied results: 49% [7], 24% [8] and 12–13% [10, 11] improvement. One review [9] concluded that reminders have no meaningful effect. Sutherland et al. [12] conducted a study similar to the present study, implementing the same modifications to the questionnaire, cover letter and mailing procedure, but in addition posted three reminders (two more than we did). Their response rate was significantly increased from 66 to 88%. Although the increase was likely due, at least in part, to the changes in the questionnaire and mailing procedure, the additional reminders may also have been important for increasing response rates and should be seriously considered.

## Conclusion

The implementation of five recommended changes in questionnaire and mailing procedures in quality control of nasal septal surgery did not result in a 20% point improvement in the response rate to postal questionnaires. The observed improvement of 8.7% may not justify the additional resources required to implement these five changes. Further improvement may require pre-notification and/or supplementary reminders.

## Abbreviations

VAS: visual analogue scale
NSQ: nasal surgical questionnaire
